# A cross-sectional analysis and forward projection of China’s gallbladder and biliary-tract cancer burden: Insights from the 2021 Global Burden of Disease Study

**DOI:** 10.1097/MD.0000000000051008

**Published:** 2026-10-16

**Authors:** Ke Yan, Cheng Li, Jia Ma, Yujie Liu, Youfu Tian, Jianfei Zhang

**Affiliations:** a Department of Surgical Oncology, Shaanxi Provincial People’s Hospital, Xi’an, Shaanxi, People’s Republic of China.

**Keywords:** Burden of disease, China, Gallbladder and biliary tract cancer (GBTC), Incidence, Prevalence

## Abstract

Gallbladder and biliary tract cancer (GBTC) remains highly lethal. We examined 32-year epidemiological trends in China and projected outcomes to 2050. Using 2021 Global Burden of Disease (GBD) data, we extracted annual GBTC incident and prevalent cases, deaths, and disability-adjusted life years (DALYs) for 1990 to 2021, stratified by sex, age, and socio-demographic index (SDI). The age-standardized rates (ASRs) were calculated, and ARIMA models forecast 2050 incidence and mortality. Globally, GBTC risk rises after age 60 and correlates with SDI (*R* = 0.588). In China, cases increased from 17,077 in 1990 to 51,720 in 2021, raising the age-standardized incidence rate (ASIR) from 2.04 to 2.49/100,000 (+0.50% annually). Prevalent cases increased to 79,688 (age-standardized prevalence rate (ASPR) 3.77/100,000). Conversely, ASMR fell from 2.32 to 1.85/100,000 (−0.75%), and DALYs declined from 51.3 to 40.4/100,000 (−0.87%), driven by fewer years of life lost (YLL). Males drove incidence and prevalence. Projections suggest male incidence will keep rising until 2050, while female rates are projected to plateau; male mortality stays stable, and female mortality continues a gradual decline. Aging and rising SDI inflate GBTC incidence, yet improved care compresses mortality. Males carry an increasing share. Targeted prevention and early detection are critical to curb incidence and improve survival.

## 1. Introduction

Malignancies of the bile-secreting tree-gallbladder carcinoma (GBC) and cholangiocarcinoma (CCA)^[1,2]^-share an embryonic origin in the biliary epithelium yet behave like a family of orphan diseases.^[3–5]^ Together they account for roughly one in twenty gastrointestinal cancers worldwide, a statistic that belies their lethal reputation: more than half of patients present with locally advanced or metastatic disease, only one in three tumors is amenable to curative resection, and five-year survival hovers around ten percent.^[6]^ The classic triad of vague right-sided discomfort, transient jaundice, and postprandial bloating is so easily blamed on gallstones that the first clue to cancer is often an incidental finding on pathology.^[7]^ Meanwhile, global drivers-aging populations, the epidemiological transition characterized by increasing metabolic risk factors, chronic Salmonella carriage, hepatolithiasis, and primary sclerosing cholangitis – are quietly expanding the at-risk pool, eroding the long-held label of “rare tumor.”^[7–10]^

Intrahepatic CCA has doubled in incidence in East Asia over 2 decades, whereas gallbladder cancer clusters in the Andean highlands and Northern India; peri-hilar tumors prefer men in their seventh decade, yet distal CCA strikes women earlier and more aggressively. Histologically, adenocarcinomas dominate, but signet-ring cells, squamous variants, and the presence of mixed neuroendocrine-neuroendocrine tumor components, each wired to distinct stromal niches and immune deserts, exist. This heterogeneity highlights the limitations of conventional imaging modalities: a one-millimeter mural thickening or an isolated ductular dilatation is often dismissed until symptoms declare an incurable stage. Serum CA19-9, handicapped by cholestasis-induced false surges, remains a prognostic indicator with limited clinical utility, while next-generation sequencing reveals a patchwork of FGFR2 fusions, IDH1 hotspot mutations, ERBB2 amplifications, and BAP1 loss – actionable in principle, yet frustrated by low allele frequencies and spatial heterogeneity.^[11–13]^

China, now home to one-fifth of the world’s seniors and the fastest-growing diabetic cohort, is approaching an inflection point. Modelling exercises suggest that within the next decade the country will graduate from moderate to high GBTC burden, however recent GBD-based publications have not characterized the specific, high-resolution analyses focusing on China’s unique epidemiological transition remain limited. Existing studies often aggregate global data, potentially obscuring nation-specific patterns driven by China’s rapid demographic aging and healthcare reforms. Furthermore, previous projections frequently lack accompanying uncertainty intervals relevant for local health-service planning. This study addresses these gaps by providing a comprehensive, China-centric analysis of GBTC burden from 1990 to 2021. Crucially, we move beyond mere description to quantify the divergent trajectories of incidence/prevalence versus mortality/DALYs, elucidate the emerging male predominance through detailed sex-disaggregated age-period-cohort analysis, and generate 2050 forecasts complete with robust uncertainty estimates. Our findings aim to inform targeted resource allocation within China’s evolving healthcare landscape, offering insights distinct from global overviews or purely methodological GBD investigations.

## 2. Methods & materials

### 2.1. Data provenance

Country-level GBTC estimates for 1990 to 2021 were extracted from the Global Burden of Disease (GBD) Study 2021 (version released May 2024) via the GBD Results Tool (https://vizhub.healthdata.org/gbd-results/) on March 12, 2024. Data were queried using the following parameters: GBD Cause ID 389 (“Gallbladder and biliary tract cancers”), mapped to ICD-10 codes C23-C24.9; measures included incidence, prevalence, mortality, YLL, years lived with disability (YLD), and DALYs; metrics included both counts and rates (per 100,000 population); locations comprised 204 countries and territories (China: Location ID 6); stratification was by sex (male, female) and 14 five-year age groups (0–4 to 85 + years). Age-standardized rates were downloaded directly as pre‑computed variables from the GBD Results Tool. All extraction procedures adhered to the GATHER guidelines for health estimates reporting.

### 2.2. Data refinement

Prior to analysis, we reconciled the nested GBD cause hierarchy (Levels 3–5) to ensure comprehensive inclusion of all GBTC-related ICD-10 codes. GBD 2021 does not provide separate estimates for histological subtypes (e.g., adenocarcinoma, squamous cell carcinoma); therefore, this study reports aggregate GBTC burden only. GBD estimates inherently utilize the 2021 revision of the United Nations World Population Prospects as denominators and apply the GBD global standard population (2013 GBD standard) for age-standardization. Accordingly, no secondary rescaling or mathematical transformation of raw GBD counts or rates was performed. Uncertainty intervals (95% UI), derived by the GBD modeling team from 1000 posterior draws of the Bayesian meta‑regression model (DisMod-MR 2.1), were retained throughout all analyses. Age-standardized rates (ASIR, ASPR, ASMR, age-standardized DALY rate) were computed by the GBD modeling team using the GBD global standard population (2013 GBD standard) and downloaded directly from the GBD Results Tool to ensure consistency with GBD reporting conventions.

### 2.3. Trend interrogation

Temporal trends in GBTC burden were analyzed using the Joinpoint Regression Program (version 4.9.0.1, National Cancer Institute, USA).^[14]^ A log-linear model was fitted to the annual ASRs according to the formula ln (y)=α+βx+ε, where y represents the ASR and × denotes the calendar year. Natural logarithmic transformation was applied to stabilize variance. The maximum number of joinpoints was restricted to three, permitting up to 4 distinct trend segments across the 1990 to 2021 period. Empirical Bayesian variance adjustment was employed to account for over-dispersion in the GBD estimates; no additional autocorrelation structures were imposed. The optimal number of joinpoints was determined via a Monte Carlo permutation test employing 4999 simulations, with statistical significance set at *P* < .05.

Two summary measures were reported. Percent Change (PC) quantified the relative difference in the absolute number of cases, deaths, or DALYs between 2 specified calendar years. Average Annual Percent Change (AAPC)^[15]^ described the estimated annual percentage change in the age-standardized rate over a fixed period, derived by weighting each segment’s annual percent change by its length. Both PC and AAPC are reported with 95% confidence intervals (CIs), calculated using the Joinpoint software. It is important to distinguish these statistical CIs from the 95% uncertainty intervals (UIs) provided by the GBD collaboration, which reflect modeling uncertainty from 1000 posterior draws of the Bayesian meta-regression (DisMod-MR 2.1) and were retained throughout the analysis but not used for inferential testing of trends.

### 2.4. Socio-demographic index

The socio-demographic index (SDI) is a composite score (0 = lowest, 1 = highest development) derived by GBD from lag-distributed income per capita, mean years of schooling, and total fertility rate. Countries are assigned to quintiles (low, low–middle, middle, high–middle, high) that remain fixed across the study period to permit comparative trending.

### 2.5. Forward projection (2022–2050)

Separate ARIMA(p, d, q) models were fitted to each national age-standardized incidence and mortality series.^[16]^ Stationarity was assessed using the Augmented Dickey–Fuller (ADF) test; first‑order differencing (d = 1) was applied where ADF *P* > .05. Model orders (p,d,q) were identified via examination of the autocorrelation function (ACF) and partial autocorrelation function (PACF), and optimal specifications were selected based on the lowest Akaike Information Criterion corrected (AICc) for small samples on a 70% training split. Final models were refitted on the full series. Residual diagnostics showed no significant autocorrelation (Ljung–Box test *P* > .05) and approximate normality. The exact GBD release version, access date, cause and location IDs, ICD-10 mapping, measure/metric types, age-group and sex categories, and the standard population used for age-standardization are listed in Table S1, Supplemental Digital Content 1.

## 3. Results

### 3.1. Age-stratified GBTC burden, 1990–2021: global picture and regional contrasts

First, age-specific GBTC incidence worldwide and by region was examined. The results show a sharp rise after age 70 in virtually every area. High-income Asia Pacific leads (221.1 per 100,000), followed by Australasia (71.7 per 100,000), Southern Latin America (60.7 per 100,000), and Western Europe (50.8 per 100,000); these high rates probably reflect both aging populations and better diagnosis. Low-income African regions record the lowest burden, yet underdiagnosis is likely (Fig. 1A). This provides visual evidence for allocating public-health resources, identifying priority groups for early intervention, and conducting cross-regional comparative studies.

**Figure 1.**
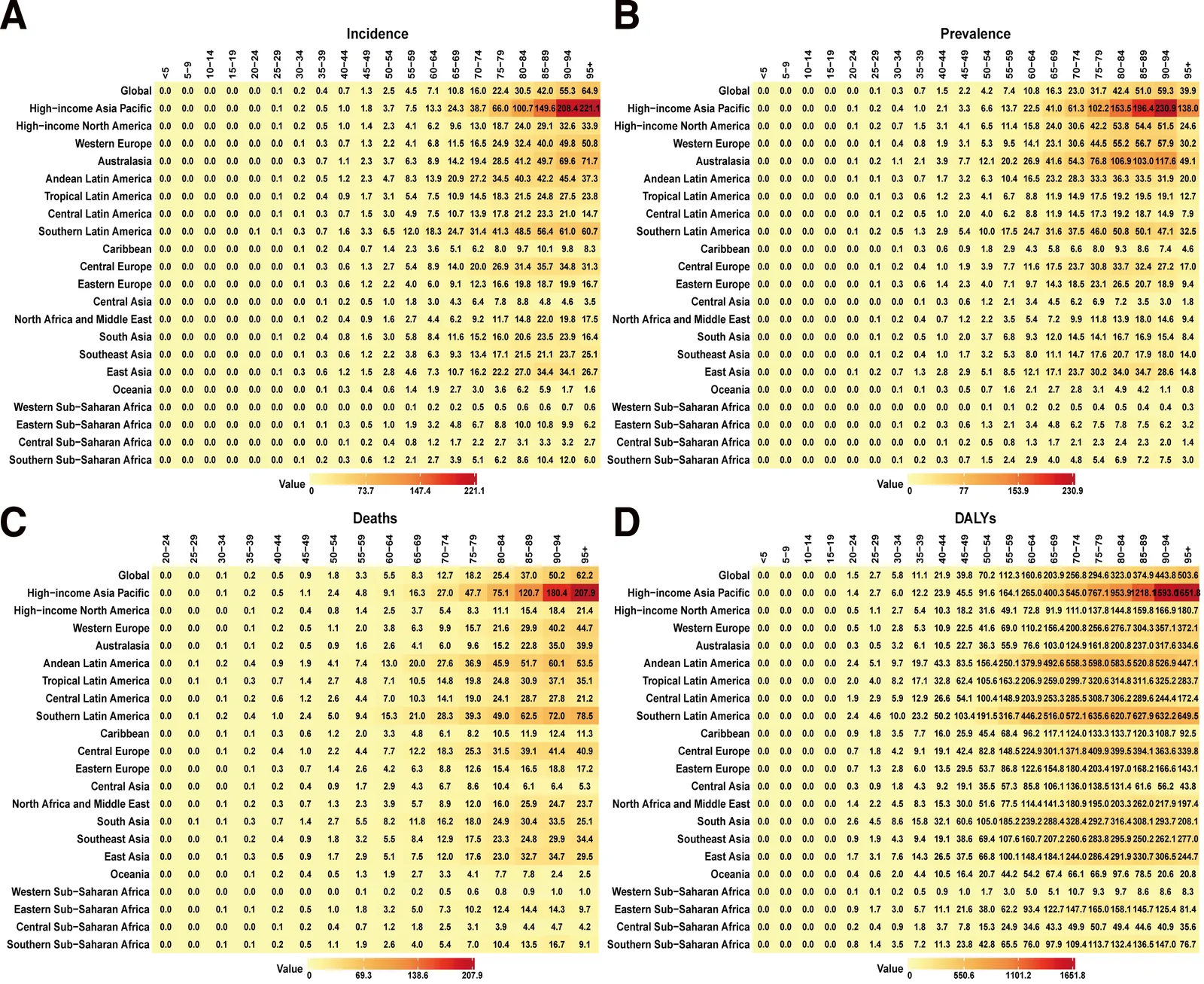
Global and regional age-specific GBTC incidence, prevalence, mortality, and DALYs. (A) Global and regional age-specific GBTC incidence, (B) Global and regional age-specific GBTC prevalence, (C) Global and regional age-specific GBTC mortality, (D) Global and regional age-specific GBTC DALYs.

Age-specific prevalence follows a similar pattern: a clear upswing begins at 60 years and peaks at 80+. Global prevalence is 39.9 per 100,000, whereas High-income Asia Pacific reaches 138.0 per 100,000 (the highest). In contrast, Western Sub-Saharan Africa shows the lowest figure, only 0.3 per 100,000. The heavy burden in high-income countries probably stems from population aging, well-established chronic-disease management, and stronger diagnostic capacity (Fig. 1B).

Mortality curves climb markedly from age 50 and peak in those aged 95+. High-income Asia Pacific again tops the list, at 207.9 per 100,000, while Southern Latin America reaches 78.5 per 100,000 in the oldest old. Andean Latin America, Western Europe, and Central Europe follow, all exceeding 40 per 100,000 in the highest age band. Notably, although Australasia has a high incidence, its mortality is comparatively modest (Fig. 1C).

DALYs, the core metric of disease burden, integrate both death (YLL) and disability (YLD)^[17,18]^; higher values indicate greater overall harm to population health. Figure 1D presents age-specific GBTC DALYs worldwide and by region. They rise exponentially with age; among those 80+, High-income Asia Pacific records the highest DALYs (1651.8 per 100,000), followed by Southern Latin America (649.5 per 100,000). Andean Latin America and South Asia rank next in succession.

### 3.2. SDI versus age-standardized incidence and DALYs

The age-standardized rate (ASR) removes the confounding effect of different population pyramids, allowing direct comparison of underlying disease risk.^[19]^ When 2021 ASIR is plotted against SDI for 204 countries, a positive ecological association emerges: high-SDI nations (e.g., Japan, Korea, Germany, Italy) exhibit ASIR values between 5 and 7.5 per 100,000, whereas low-SDI countries (e.g., India, Kenya, Niger) register < 1 per 100,000. The Pearson correlation coefficient is 0.588 (*P* < .001), suggesting that higher SDI co-occurs with elevated detected GBTC risk, potentially reflecting enhanced diagnostic capacity and risk factor exposure in more developed settings. The same upward trajectory is latent in the global south: as SDI rises, ASIR may increase, underscoring the need for early implementation of upstream surveillance (Fig. 2A).

**Figure 2.**
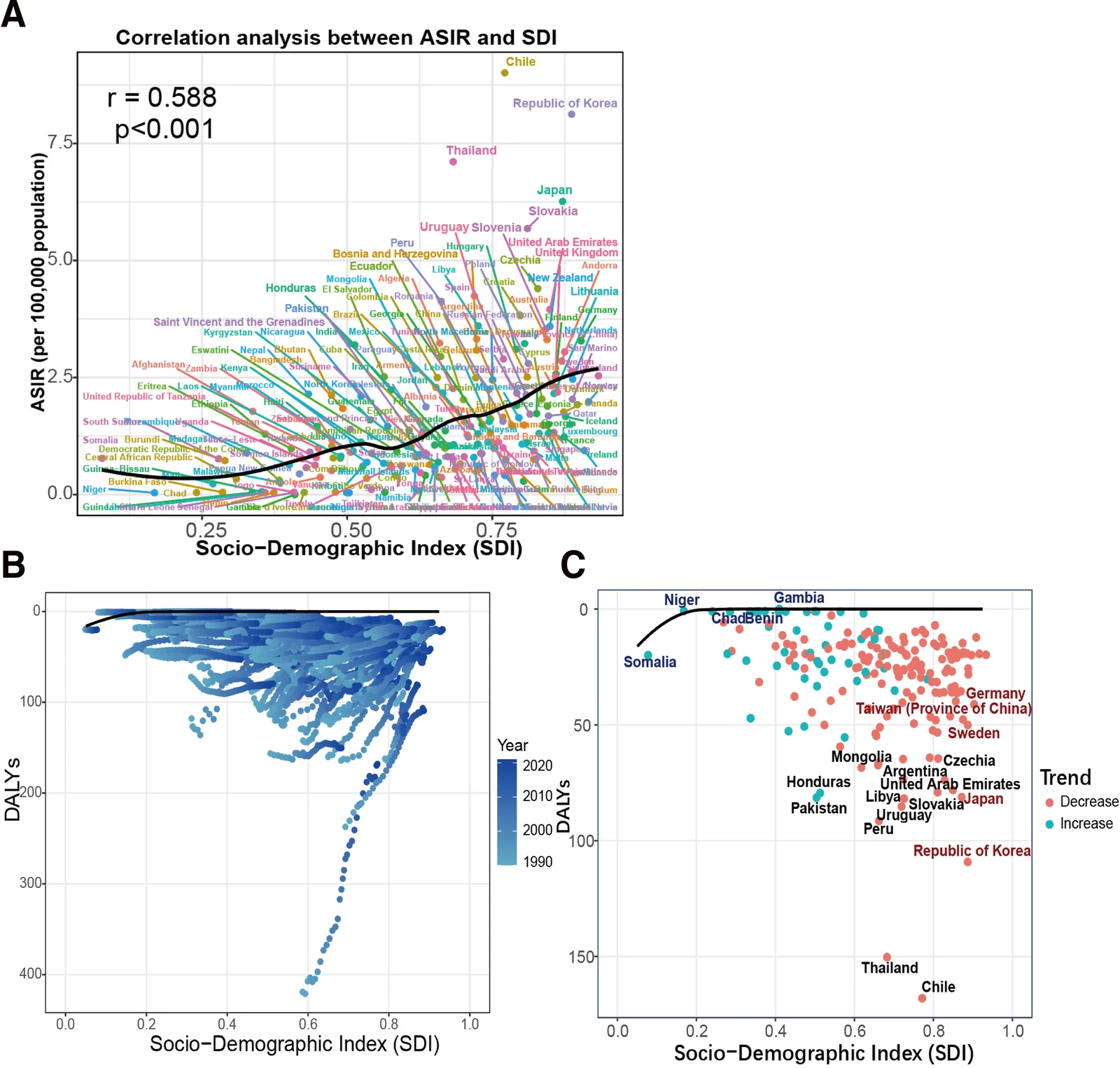
Correlation analyses of SDI with age-standardized GBTC incidence and DALYs. (A) SDI versus age-standardized incidence rate of GBTC. (B) Scatter trend plot of SDI versus DALYs. (C) Country-specific scatter trend plots of SDI versus DALYs worldwide.

The relationship with DALYs moves in the opposite direction. Each national cloud in Figure 2B darkens with time, and for most countries, the path runs downward: rising SDI is accompanied by falling DALYs. By 2021, the average global burden had settled near 100 DALYs per 100,000 at SDI ≈ 0.9. A closer look at individual country trends shows that Niger, Somalia, Chad, and Benin – all with low SDI – likewise record low GBTC DALYs. However, these low estimates likely reflect underdiagnosis, incomplete cancer registration, and limited death certification quality in these regions, rather than a true absence of disease burden. In contrast, Chile, Thailand, the Republic of Korea, Japan, Sweden, Taiwan (Province of China), and Germany experienced rising DALYs as their SDI increased (Fig. 2C).

### 3.3. Evolution of GBTC disease burden in China (1990–2021)

The analysis then examined China’s GBTC burden from 1990 to 2021. Compared with 1990, the number of incident cases tripled, and the age-standardized rate increased markedly. In the male subgroup, both case numbers and the age-standardized rate rose significantly: PC reached 29% and the average AAPC was 1%, indicating an accumulation of risk factors in men. In the female subgroup, case numbers increased, but the age-standardized rate showed almost zero growth, implying that the female incidence was driven by population aging rather than a true rise in risk; thus, the increase in male incidence is the main source of the national GBTC burden.

Regarding prevalence, the absolute number of prevalent cases in 2021 (79,688) was more than 4 times the 1990 figure (17,980). The age-standardized prevalence rate (ASPR) rose from 2.18 to 3.77 per 100,000, representing a PC of 72.9% (AAPC: 2.04%, 95% CI: 1.85–2.22), indicating a rapid increase in the underlying prevalence burden.

Regarding deaths, the 2021 count was roughly double the 1990 figure; however, as China’s population base grew during 1990 to 2021, the 2021 GBTC mortality rate was 1.85 per 100,000, a clear decrease from 2.32 per 100,000 in 1990, with PC at − 20.3% and AAPC at − 0.75%, indicating a decline in mortality. The same trend was observed in both sexes, and male mortality remained higher than female.

DALYs numbered 452,349 in 1990 and 857,504 in 2021, an obvious increase in absolute terms, yet the DALY rate fell from 52.61 per 100,000 in 1990 to 40.38 per 100,000 in 2021 (PC: −23.2%; AAPC: −0.87%). This trend may plausibly reflect the impact of economic growth and advancements in medical care, including earlier diagnosis and more effective treatments, on reducing GBTC-related disability and premature mortality. Notably, YLLs had a PC of − 23.7%, indicating a reduction in the age-standardized YLL rate per 100,000 population, whereas YLDs had a PC of + 25.50%. The increase in YLDs may reflect a combination of factors, including improved survival due to advances in clinical care, longer disease duration among prevalent cases, or refinements in GBD disability weight modeling, rather than being solely attributable to improved prognosis (details in Table 1).

**Table 1:** Incidence, prevalence, mortality, DALYs, YLDs, and YLLs of gallbladder and bile-duct cancer in China (1990–2021).

Measure	Sex	Number.1990	Rate.1990	Number.2021	Rate.2021	PC (%)	AAPC, % (95% UI)
Incidence	Male	8138 (5378, 11,476)	2.24 (1.46, 3.23)	27,797 (16,110, 38,966)	2.89 (1.69, 4)	29	1 (0.88, 1.11)
Incidence	Female	8940 (6147, 12,548)	2.19 (1.51, 3.05)	23,923 (14,698, 32,562)	2.17 (1.33, 2.95)	−0.9	−0.04 (−0.14, 0.06)
Incidence	Both	17,077 (13,003, 21,744)	2.19 (1.68, 2.79)	51,720 (35,618, 66,848)	2.49 (1.71, 3.21)	13.7	0.5 (0.4, 0.6)
Prevalence	Male	8792 (5766, 12,289)	2.22 (1.45, 3.16)	44,801 (25,705, 62,876)	4.47 (2.58, 6.19)	101.4	2.62 (2.41, 2.83)
Prevalence	Female	9188 (6276, 12,800)	2.16 (1.48, 3)	34,887 (21,146, 47,646)	3.15 (1.91, 4.3)	45.8	1.35 (1.2, 1.51)
Prevalence	Both	17,980 (13,589, 22,713)	2.18 (1.66, 2.74)	79,688 (53,151, 103,929)	3.77 (2.51, 4.91)	72.9	2.04 (1.85, 2.22)
Deaths	Male	8081 (5345, 11,612)	2.38 (1.55, 3.5)	19,525 (11,615, 27,421)	2.11 (1.26, 2.92)	−11.3	−0.34 (−0.47, −0.22)
Deaths	Female	9170 (6355, 12,939)	2.32 (1.6, 3.24)	18,309 (11,423, 24,764)	1.67 (1.04, 2.25)	−28	−1.17 (−1.29, −1.04)
Deaths	Both	17,251 (13,213, 22,143)	2.32 (1.78, 2.97)	37,833 (26,653, 49,262)	1.85 (1.29, 2.4)	−20.3	−0.75 (−0.87, −0.64)
DALYs	Male	221,390 (145,097, 311,194)	52.86 (34.73, 75.75)	459,793 (269,773, 651,240)	45.57 (26.8, 63.82)	−13.8	−0.41 (−0.51, −0.31)
DALYs	Female	230,959 (158,402, 322,844)	52.85 (36.17, 73.76)	397,711 (248,642, 540,089)	35.83 (22.37, 48.65)	−32.2	−1.36 (−1.47, −1.25)
DALYs	Both	452,349 (341,842, 581,398)	52.61 (39.99, 67.15)	857,504 (601,927, 1,121,113)	40.38 (28.21, 52.61)	−23.2	−0.87 (−0.96, −0.77)
YLDs	Male	1961 (1172, 2938)	0.53 (0.31, 0.79)	7305 (4076, 11,105)	0.75 (0.42, 1.13)	41.50	1.34 (1.2, 1.47)
YLDs	Female	2116 (1295, 3051)	0.51 (0.31, 0.74)	6069 (3496, 9090)	0.55 (0.32, 0.82)	7.80	0.24 (0.14, 0.35)
YLDs	Both	4077 (2574, 5778)	0.51 (0.33, 0.73)	13,374 (8028, 19,633)	0.64 (0.39, 0.93)	25.50	0.82 (0.71, 0.93)
YLLs	Male	219,429 (143,738, 307,988)	52.33 (34.35, 74.87)	452,488 (265,998, 642,184)	44.83 (26.41, 62.92)	−14.3	−0.43 (−0.53, −0.33)
YLLs	Female	228,843 (156,698, 320,158)	52.34 (35.76, 73.11)	391,642 (244,037, 531,928)	35.28 (21.95, 47.83)	−32.6	−1.38 (−1.49, −1.27)
YLLs	Both	448,272 (338,886, 575,981)	52.09 (39.61, 66.4)	844,130 (592,378, 1,102,409)	39.74 (27.77, 51.72)	−23.7	−0.89 (−0.98, −0.79)

“Rate” refers to age-standardized rates (ASR) based on the GBD world population standard.

AAPC = average annual percent change, DALYs = disability-adjusted life years, PC = percent change, YLDs = years lived with disability, YLLs = years of life lost.

Age-standardized indicators for China during 1990 to 2021 showed that the age-standardized incidence rate (ASIR) and age-standardized mortality rate (ASMR) fluctuated, the age-standardized prevalence rate (ASPR) rose slowly, and the DALY rate declined markedly (Figs. 3A–D). Analysis of sex-specific ASIR and ASPR revealed that male ASIR increased steadily, whereas female ASIR peaked around 2015 and then declined slowly. Therefore, the overall ASIR rise was chiefly contributed by men, and male ASIR exceeded female ASIR, with the gap widening acceleratively after 2015 (Fig. 3E). For ASPR, the overall rate trended upward; both male and female ASPR increased, male ASPR was higher than female, and the sex gap widened over time, indicating that male ASPR accumulated faster than female (Fig. 3F).

**Figure 3.**
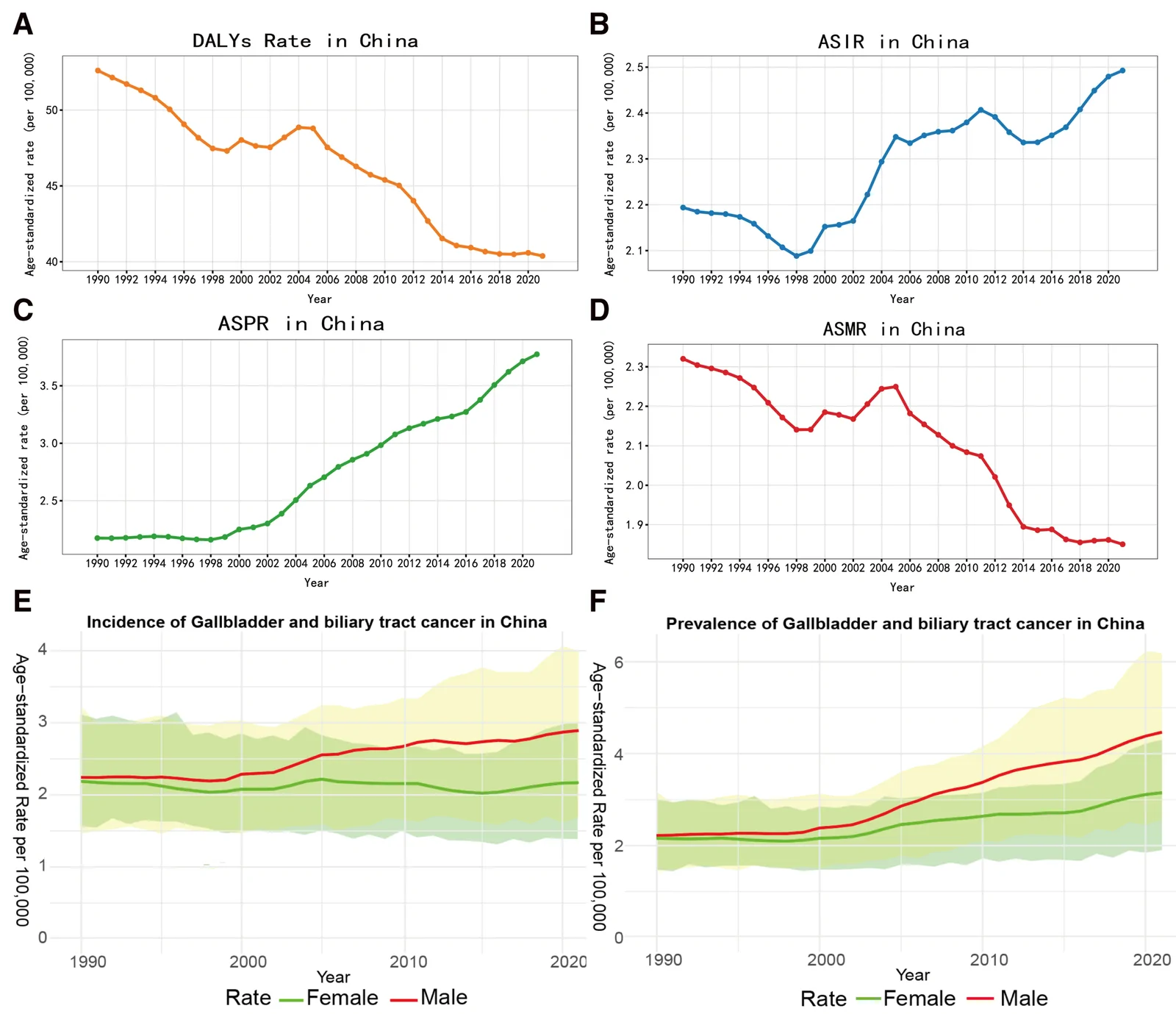
Evolution of GBTC burden in China (1990–2021). (A–D) Scatter plot of age-standardized disease-burden indicators for GBTC in China, including ASIR, ASPR, ASMR, and DALYs rate. (E) Composite plot of age-standardized incidence rate for GBTC in China. (F) Composite plot of age-standardized prevalence rate for GBTC in China.

### 3.4. Disease Burden of GBTC in Chinese Males and Females Across Age Groups

Although the absolute burden metrics in 2021 appeared broadly similar between sexes (e.g., 27,797 incident cases in males vs 23,923 in females), sex-specific analyses revealed divergent trajectories. Males exhibited a steeper average annual percent change (AAPC) in incidence (1.00% vs −0.04%) and prevalence (2.62% vs 1.35%), establishing a clear male predominance driven by accelerated growth in the male population. Incidence and prevalence plots display nonzero values before age 5, likely reflecting modeling estimates for rare early-life cases or small-number instability in the GBD statistical framework (Fig. 4A, B). Death rates remain low before 50 but surge after 70, lagging behind the incidence/prevalence curves. This mismatch suggests either “high prevalence yet low lethality” or a “delayed mortality peak,” probably attributable to prolonged survival from advances in medical care over recent decades (Fig. 4C). DALYs, the sum of YLLs and YLDs, follow a broadly similar pattern; notably, YLL bars are markedly taller than YLD bars, showing that premature death contributes far more than disability, consistent with the short clinical course and poor prognosis of GBTC (Figs. 4D–F).

**Figure 4.**
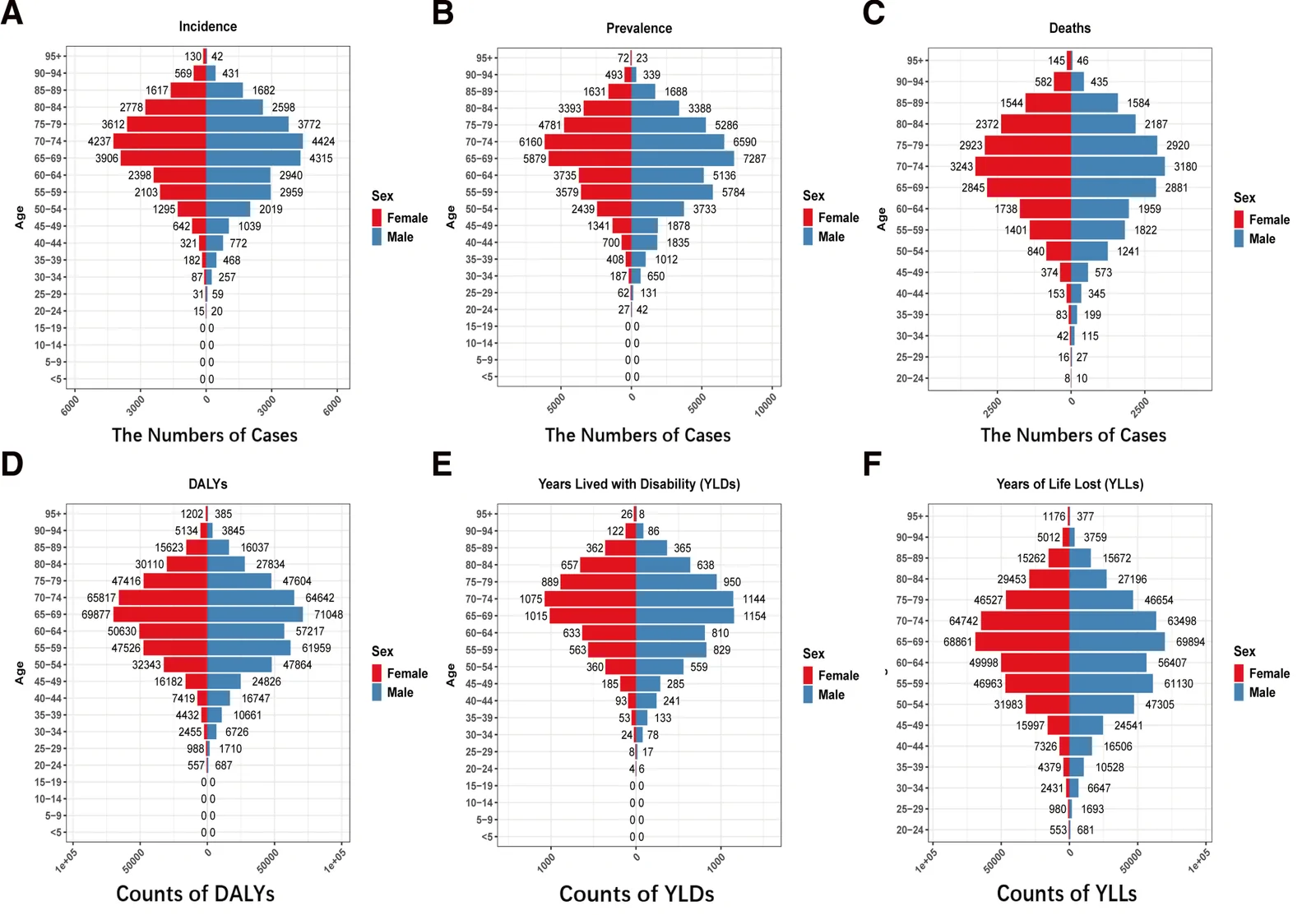
GBTC disease burden in Chinese males and females by age group. (A) Age-specific incidence of GBTC in Chinese males and females. (B) Age-specific prevalence of GBTC in Chinese males and females. (C) Age-specific deaths from GBTC in Chinese males and females. (D) Age-specific DALYs due to GBTC in Chinese males and females. (E) Age-specific YLLs due to GBTC in Chinese males and females. (F) Age-specific YLDs due to GBTC in Chinese males and females.

### 3.5. Age-related trends of GBTC burden in Chinese males and females

We next compared sex-specific indicators. All metrics-incidence, prevalence, mortality, DALYs, YLDs and YLLs-rose with age in both sexes. After age 70, however, the curves for males surged, widening the gap with females. This male predominance persisted until about age 85, after which the gap narrowed again as the male indicators fell sharply. The findings highlight the strong age dependence of GBTC burden in China and the disproportionately high share borne by men (Figs. 5A–F).

**Figure 5.**
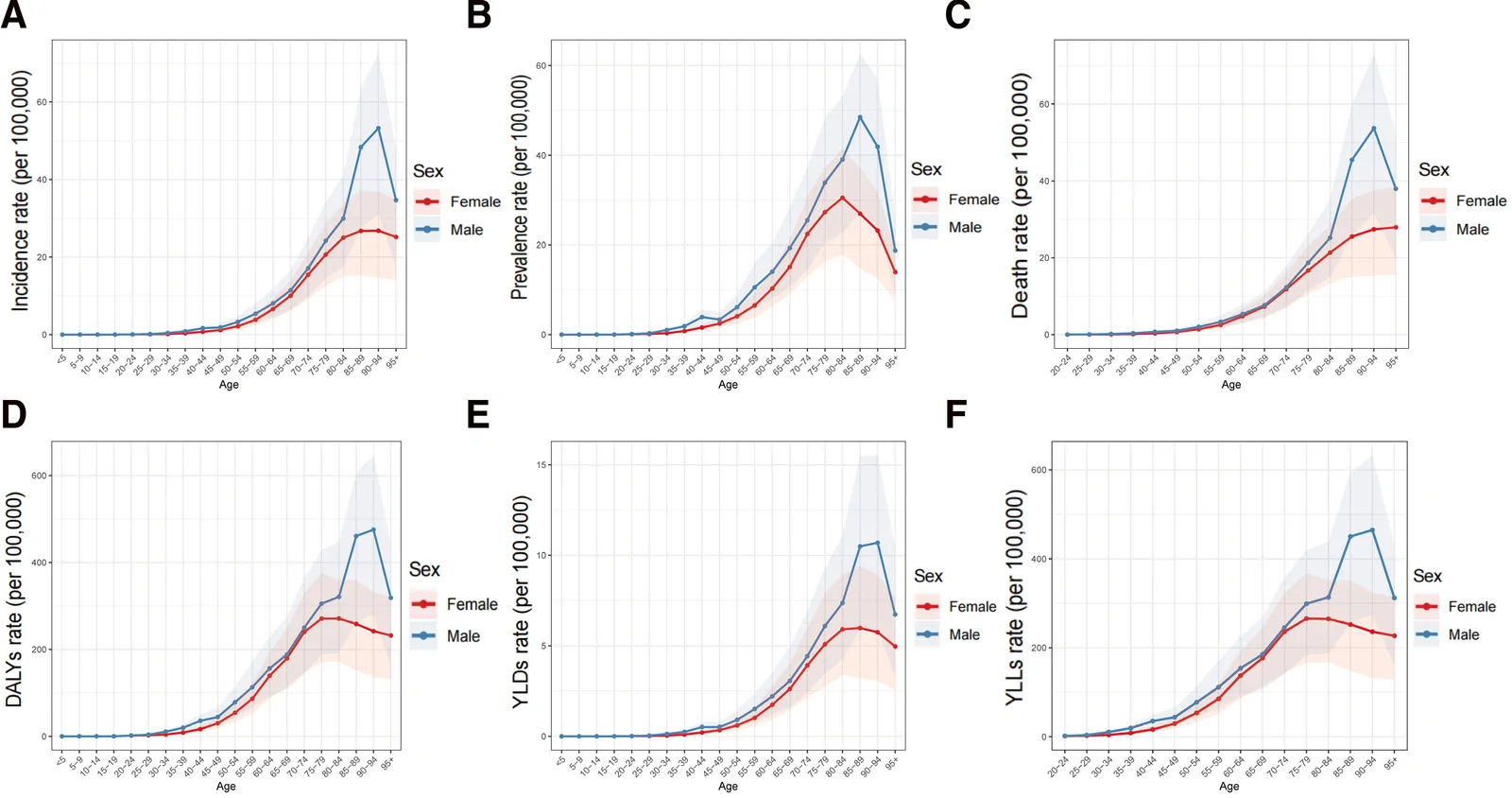
Age-related trajectories of GBTC burden in Chinese males and females. (A) Age-specific incidence of GBTC in Chinese males and females. (B) Age-specific prevalence of GBTC in Chinese males and females. (C) Age-specific deaths from GBTC in Chinese males and females. (D) Age-specific DALYs due to GBTC in Chinese males and females. (E) Age-specific YLDs due to GBTC in Chinese males and females. (F) Age-specific YLLs due to GBTC in Chinese males and females.

### 3.6. Projected age-standardised incidence and prevalence of GBTC in China to 2050

We then forecast the GBTC burden for the world, high-, middle-, and low-SDI regions, and China over 2022 to 2050. Globally, the age-standardized incidence rate (ASIR) is projected to keep rising in males but to decline in females; the age-standardized mortality rate (ASMR) is expected to fall in both sexes (Fig. S1A–D, Supplemental Digital Content 2).

In high-SDI countries, male ASIR will plateau at today’s level, female ASIR will drop, and ASMR will follow the same downward track seen worldwide (Fig. S1E–H, Supplemental Digital Content 2). In middle-SDI settings, male ASIR will continue its ascent, female ASIR will stabilize, male ASMR will remain roughly flat, and female ASMR will keep decreasing (Fig. S1I–L, Supplemental Digital Content 2). In low-SDI regions, both ASIR and ASMR are set to increase for men and women alike (Fig. S1M–P, Supplemental Digital Content 2).

For China, models indicate that male ASIR will keep climbing through 2050 (Fig. 6A), whereas female ASIR will decline until ~2030 and then level off (Fig. 6B). ASMR projections show that male rates will stay essentially constant over 2022 to 2050 (Fig. 6C), while female rates will continue their downward trend (Fig. 6D). Numerical projections indicate that male ASIR is forecast to rise from 2.49/100,000 in 2021 to 2.71 (95% PI: 2.34–3.08) by 2030, 3.05 (95% PI: 2.41–3.79) by 2040, and 3.42 (95% PI: 2.48–4.51) by 2050. Female ASIR is projected to decline from 2.49/100,000 to 2.18 (95% PI: 1.89–2.47) by 2030 and stabilize at 2.09 (95% PI: 1.62–2.63) by 2050. Male ASMR is expected to remain stable at approximately 1.85/100,000 (2050: 1.82, 95% PI: 1.41–2.28), whereas female ASMR is forecast to decline from 1.85 to 1.43 (95% PI: 1.08–1.84) by 2050. Absolute incident cases in China are projected to reach 63,400 (95% PI: 54,800–72,600) by 2030 and 78,200 (95% PI: 62,100–95,700) by 2050 (Table S2, Supplemental Digital Content 3).

**Figure 6.**
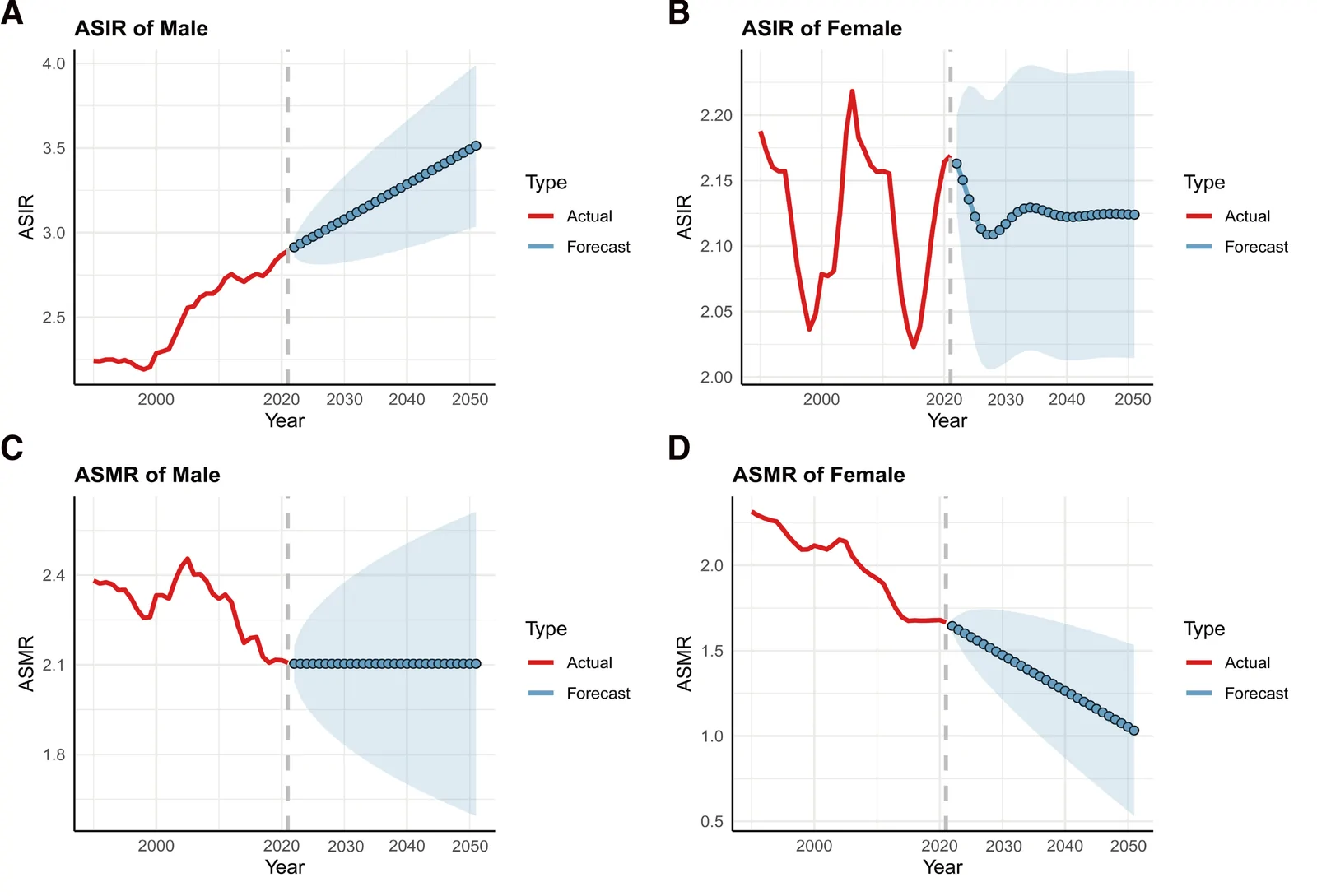
Projected age-standardized incidence and mortality rates of GBTC in China, 2022–2050. (A) Projected trend of GBTC ASIR for Chinese males. (B) Projected trend of GBTC ASIR for Chinese females. (C) Projected trend of GBTC ASMR for Chinese males. (D) Projected trend of GBTC ASMR for Chinese females.

## 4. Discussion

Drawing on the open-access Global Burden of Disease Study 2021, we profiled worldwide GBTC burden, dissected its distribution in China, and forecast the country’s disease trajectory from 2022 to 2050. Over the past 3 decades global GBTC burden has declined overall, yet every region shows a pronounced age gradient: cases, prevalence, deaths, and DALYs begin to climb steeply after age 50 and accelerate with advancing age-mirroring the pattern of most malignancies and reflecting population aging.^[20,21]^ In low- and lower-middle-income countries, however, incidence is still rising, with males carrying the heaviest load, underscoring the urgent need for targeted interventions.

Unlike many cancers, GBTC ASIR increases with SDI (*R* = 0.588). High-SDI settings record the highest incidence—probably because greater prosperity favors obesity, dyslipidemia, cholelithiasis, and better case detection-whereas low-SDI countries, despite lower incidence, suffer disproportionate mortality. Yet DALYs per 100,000 fall as SDI rises: gallbladder cancer five-year survival reaches 25% in high-SDI countries versus < 5% in low-SDI nations, and median survival in the United States (14 months) is 4.7-fold that in Nigeria (3 months).^[22]^ Late diagnosis and limited access to effective therapy in low-SDI settings translate into high YLLs; hence, these regions should invest in early detection and treatment capacity rather than dismissing GBTC as a “disease of affluence.” Conversely, high-SDI countries should prioritize the management of metabolic risk factors (e.g., obesity, diabetes) and the systematic identification of high-risk subgroups, building on existing clinical guidelines for GBTC surveillance.

Projections for the next 3 decades indicate that global male ASIR will rise-driven mainly by lower-middle-SDI regions-while female ASIR will decline, led by high-SDI countries. Both sexes are expected to record falling ASMR, again led by high-SDI nations, highlighting the complex, multi-dimensional influence of SDI on GBTC burden.

In China, incidence and prevalence rose between 1990 and 2021, potentially driven by rising SDI, increased exposure to metabolic risk factors, and improved diagnostic sensitivity, whereas mortality and DALY rates declined. This pattern may hypothetically reflect enhancements in healthcare access and quality, though causal mechanisms cannot be established from ecological data. As SDI continues to climb, China is likely to face a shifting burden: mortality may fall further while prevalence and DALYs keep rising—a dynamic, predictable epidemiologic transition.

After age 70 Chinese men experience sharp surges in incidence, prevalence, mortality, DALYs, YLDs, and YLLs, pulling away from women. Women’s rates plateau and begin to decline about 5 years earlier than men’s, and the subsequent drop is gentler, whereas men’s curves turn downward more abruptly-consistent with sex-specific life-expectancy patterns.

Forecasts for 2022 to 2050 suggest that Chinese male ASIR is projected to continue rising; female ASIR is forecast to fall and then stabilize; male ASMR is expected to plateau at present levels; and female ASMR is projected to decline-trends that align with projections for middle-SDI regions and reflect ongoing demographic aging and incremental improvements in care. The consistently lower counts of cases, prevalence, deaths, and DALYs among Chinese women, together with predicted future declines in female ASIR and ASMR, argue for sex-specific resource allocation that favors screening, diagnosis, and treatment of men.

Several limitations should be acknowledged. First, all findings are contingent upon GBD 2021 input data and its modeling assumptions. Although the GBD statistical methodology is rigorous, estimates rely heavily on data sources that vary considerably in quality across regions. Discrepancies with local epidemiological surveys are thus possible, particularly in low-SDI countries where under-ascertainment due to incomplete cancer registration, limited pathological diagnostic capacity, and low death certification completeness may lead to an underestimation of the true burden. Additionally, the GBD framework aggregates gallbladder cancer with intrahepatic, perihilar, and distal cholangiocarcinomas under the umbrella term “gallbladder and bile-duct cancer” (GBTC). This level of disaggregation is insufficient to characterize the distinct epidemiological profiles, risk factors, and clinical outcomes of these anatomically separate malignancies. Potential misclassification arising from ambiguities in ICD-10 coding (C23 vs C24) further compounds this limitation.

Second, the inclusion of the COVID-19 pandemic period (2020–2021) introduces potential biases. Pandemic-related disruptions in healthcare-seeking behavior, reductions in endoscopic and radiological screening, and the reallocation of medical resources may have temporarily suppressed incident case ascertainment and altered mortality patterns in the final year of analysis. While the GBD modeling framework attempts to adjust for such discontinuities, these short-term perturbations may slightly attenuate the absolute burden estimates for 2021.

Third, the ecological design of this study precludes causal inference and is susceptible to ecological fallacy. Associations observed at the population level (e.g., between SDI and ASIR) cannot be extrapolated to individual patients. While we hypothesize that metabolic risk factors drive the observed trends, this study did not perform a comparative risk assessment; thus, we cannot quantify the specific contributions of obesity, diabetes, or other exposures versus genetic or environmental determinants.

Fourth, projections to 2050 should be interpreted with extreme caution. A 29-year forecast based on 32 annual observations is intrinsically uncertain and does not account for potential future disruptions such as large-scale screening programs, novel therapeutics, or abrupt shifts in metabolic risk-factor prevalence. Modeled trajectories therefore represent plausible scenarios rather than deterministic certainties. Notably, the apparent plateau in the male ASMR projection reflects point estimates derived from the Bayesian age-period-cohort (BAPC) model. The wide uncertainty intervals (e.g., 0.82–2.50 per 100,000 for 2050) indicate substantial stochastic uncertainty inherent in long-term forecasting. This flattening does not definitively imply a stabilization of underlying risk; rather, it reflects the model’s inability to identify a strong directional trend amidst historical volatility and data constraints.

Finally, while the absolute counts and age-specific projections offered herein provide a macroscopic overview of the evolving GBTC burden, they should not be used in isolation for micro-level clinical resource allocation. Effective health-service planning necessitates the integration of these national trends with local demographic data, healthcare infrastructure audits, and economic evaluations. Future subnational analyses leveraging provincial cancer registries are required to translate these epidemiological insights into precise, actionable policy recommendations.

## 5. Conclusion

Aging and rising SDI fuel China’s expanding GBTC incidence; falling mortality and DALYs mark better care. Incidence is projected to rise through 2050, with males bearing the brunt. Focus must shift to primordial prevention of metabolic risk factors, targeted early detection strategies informed by clinical evidence, and translational research to improve outcomes.

## Acknowledgments

First, we thank the editors and reviewers of this journal for their contributions to our study. We also acknowledge the data and analytical support provided by the official sources of the following databases and tools: the R Development Core Team and the Global Burden of Disease (GBD) Study. Youfu Tian made significant contributions to the early stages of this analysis and was originally designated as a co-corresponding author alongside Jianfei Zhang. However, in compliance with the journal’s policy permitting only a single corresponding author, we hereby specifically acknowledge his contribution.

## Author contributions

**Data curation:** Ke Yan, Jia Ma, Youfu Tian.

**Investigation:** Ke Yan, Cheng Li, Yujie Liu.

**Methodology:** Ke Yan.

**Resources:** Ke Yan, Jia Ma, Yujie Liu.

**Software:** Ke Yan, Cheng Li, Jianfei Zhang.

**Validation:** Ke Yan, Cheng Li, Jia Ma, Yujie Liu.

**Visualization:** Ke Yan, Cheng Li, Jia Ma, Yujie Liu, Jianfei Zhang.

**Writing – original draft:** Ke Yan, Cheng Li.

**Writing – review & editing:** Ke Yan, Cheng Li, Jia Ma, Yujie Liu, Youfu Tian, Jianfei Zhang.

**Supervision:** Cheng Li, Jia Ma, Yujie Liu.

**Formal analysis:** Jia Ma, Youfu Tian, Jianfei Zhang.

**Conceptualization:** Youfu Tian.

**Funding acquisition:** Youfu Tian.

**Project administration:** Youfu Tian.










