# Supplementary material for: A cross-sectional analysis and forward projection of China’s gallbladder and biliary-tract cancer burden: Insights from the 2021 Global Burden of Disease Study
Source: Medicine (Baltimore). 2026 Oct 16;105(42):e51008. doi: 10.1097/MD.0000000000051008 (PMC13680927; doi:10.1097/MD.0000000000051008)
Supplement: Supplementary file 1 [file medi-105-e51008-s001.docx]

**Table S1.** Supplementary methods & GBD metadata

# **S1.1 GBD 2021 Data Extraction Parameters**

All extraction parameters for the Global Burden of Disease (GBD) Study 2021 are documented below. These parameters ensure full reproducibility of the data retrieval process.

| Parameter | Specification |
| --- | --- |
| GBD version | GBD Study 2021 (released May 2024) |
| Date of access | 12 March 2024 |
| Cause name | Gallbladder and biliary tract cancers |
| GBD Cause ID | 389 |
| ICD-10 mapping | C23, C24, C24.0, C24.1, C24.8, C24.9 |
| Measures | Incidence, Prevalence, Deaths, DALYs, YLLs, YLDs |
| Metrics | Counts; Rates (per 100,000) |
| Locations | 204 countries (China: Location ID 6) |
| Age groups | 14 groups: 0–4, 5–9, …, 80–84, 85+ years |
| Sex | Male, Female |
| Standard population | GBD global standard population (2013 GBD standard) |
| Uncertainty intervals | 95% UI from 1,000 posterior draws |
| Software platform | GBD Results Tool (https://vizhub.healthdata.org/gbd-results/) |

# **S1.2 Variable Definitions & Age-Standardisation**

The following variables were used throughout the analysis. All age-standardised rates were pre-computed by the GBD modelling team and downloaded directly from the GBD Results Tool.

• No secondary rescaling of GBD counts was performed; GBD rates already use the 2021 UN World Population Prospects as denominators.

• The prevalence metric refers to GBD point prevalence (all-age, mid-year estimate), not period prevalence.

• 95% UIs reflect GBD modelling uncertainty; 95% CIs reflect uncertainty from our statistical procedures.

| Variable | Definition |
| --- | --- |
| ASIR | Age-standardised incidence rate (per 100,000), computed by GBD using 2013 GBD standard population |
| ASPR | Age-standardised prevalence rate (per 100,000), computed by GBD using 2013 GBD standard population |
| ASMR | Age-standardised mortality rate (per 100,000), computed by GBD using 2013 GBD standard population |
| Age-standardised DALY rate | Disability-adjusted life-years per 100,000, age-standardised to GBD 2013 standard |
| Prevalence metric | GBD point prevalence (all-age, mid-year), not period prevalence |
| SDI | Socio-demographic index (0–1): composite of lag-distributed income, mean schooling, total fertility |
| 95% UI | GBD uncertainty interval from 1,000 Bayesian posterior draws (DisMod-MR 2.1) |
| 95% CI | Confidence interval from our statistical procedures (Joinpoint / ARIMA / Prophet) |

# **S1.3 Statistical and Modelling Methods (Detailed)**

**S1.3a. Joinpoint Regression Parameters**

| Parameter | Specification |
| --- | --- |
| Software | Joinpoint Regression Program v4.9.0.1 (NCI, USA) |
| Model form | Log-linear: ln(y) = α + βx + ε |
| Transformation | Natural logarithm of age-standardised rates |
| Max joinpoints | 3 (up to 4 trend segments, 1990–2021) |
| Variance adjustment | Empirical Bayesian (over-dispersion) |
| Autocorrelation | None imposed beyond GBD UIs |
| Permutations | 4,999 Monte Carlo simulations |
| Significance level | P < 0.05 |
| AAPC reference | Kim et al. (Ref. 14) — Joinpoint CI estimation |

**S1.3b. ARIMA Model Specifications**

Stationarity was assessed using the Augmented Dickey–Fuller (ADF) test. First-order differencing (d = 1) was applied where ADF p > 0.05. Model orders (p,d,q) were identified via ACF/PACF examination and selected by minimum AICc on a 78% training split (1990–2014). Final models were refitted on the full 1990–2021 series. Residual diagnostics (Ljung–Box test, p > 0.05; Shapiro–Wilk normality) confirmed model adequacy.

**S1.3c. Prophet Model Specifications**

Prophet models were fitted with linear trend, yearly seasonality enabled, weekly seasonality disabled (annual data), and changepoint prior scale = 0.05. Interval width was set to 0.95 for 95% uncertainty intervals. Posterior sampling generated medians and 95% UIs.

**S1.3d. Model Comparison & Selection (China)**

Both ARIMA and Prophet were evaluated on the held-out 2015–2021 validation set using RMSE, MAE, MAPE, and prediction-interval coverage. ARIMA outperformed Prophet on all four metrics for every outcome; therefore, ARIMA forecasts are presented in the main text (Fig. 6), with Prophet results archived here.

| Outcome | Model | ARIMA (p,d,q) | ADF Stat (p) | Prophet Prior | RMSE | MAE | MAPE (%) |
| --- | --- | --- | --- | --- | --- | --- | --- |
| ASIR Male | ARIMA | (1,1,1) | −4.21 (<0.01) | – | 0.087 | 0.071 | 3.42 |
| ASIR Female | ARIMA | (0,1,1) | −3.98 (<0.01) | – | 0.074 | 0.062 | 2.98 |
| ASMR Male | ARIMA | (0,1,1) | −4.05 (<0.01) | – | 0.069 | 0.058 | 3.10 |
| ASMR Female | ARIMA | (1,1,0) | −3.87 (<0.01) | – | 0.063 | 0.054 | 2.87 |
| Prophet (all) | – | – | – | 0.05 | 0.092 | 0.074 | 3.68 |

# **S1.4 Sensitivity Analyses**

Four sensitivity scenarios were conducted to assess the robustness of trend and forecast estimates.

| No. | Scenario | Result |
| --- | --- | --- |
| 1 | Varied train/test splits (60/40, 80/20) | Core trajectories unchanged; ARIMA consistently outperformed Prophet |
| 2 | Adjusted Prophet changepoint prior (0.01–0.5) | Prior 0.05 yielded optimal balance between fit and over-fitting |
| 3 | Excluded data-sparse small island nations | Global trends and SDI gradients preserved |
| 4 | Different max joinpoint settings (2 vs. 3 vs. 4) | Three joinpoints captured all meaningful inflection points |

# **S1.5 Software & Reproducibility**

• R version 4.4.0 (R Foundation for Statistical Computing)

• R packages: forecast, prophet, tidyverse, ggplot2

• Joinpoint Regression Program v4.9.0.1 (NCI, USA)

• GBD Results Tool (https://vizhub.healthdata.org/gbd-results/)

Full R code and processed datasets are available at [Zenodo/GitHub DOI: XXX] to ensure reproducibility.

*Note: Replace [Zenodo/GitHub DOI: XXX] with the actual repository DOI before submission. All GBD estimates are publicly available and were used in accordance with IHME data access policies.*
