# Supplementary material for: A cross-sectional analysis and forward projection of China’s gallbladder and biliary-tract cancer burden: Insights from the 2021 Global Burden of Disease Study
Source: Medicine (Baltimore). 2026 Oct 16;105(42):e51008. doi: 10.1097/MD.0000000000051008 (PMC13680927; doi:10.1097/MD.0000000000051008)
Supplement: Supplementary file 2 [file medi-105-e51008-s003.docx]

**Table S2.** Projected GBTC burden in China (2030, 2040, 2050) with 95% prediction intervals from ARIMA forecasts.

| Year | Metric | Male (95% PI) | Female (95% PI) | Total (95% PI) | Model / Notes |
| --- | --- | --- | --- | --- | --- |
| 2030 | ASIR (/100k) | 2.71 (2.34–3.08) | 2.18 (1.89–2.47) | 2.45 (2.18–2.74) | ARIMA(1,1,1) / (0,1,1) |
| 2030 | ASPR (/100k) | 4.12 (3.65–4.62) | 3.55 (3.08–4.06) | 3.84 (3.41–4.29) | ARIMA(1,1,1) / (0,1,1) |
| 2030 | ASMR (/100k) | 1.86 (1.52–2.24) | 1.61 (1.28–1.99) | 1.74 (1.43–2.09) | ARIMA(0,1,1) / (1,1,0) |
| 2030 | Incident cases | 38,200 (32,900–43,600) | 25,200 (21,800–28,700) | 63,400 (54,800–72,600) | ARIMA forecast |
| 2030 | Prevalent cases | 58,100 (51,400–65,200) | 41,000 (35,500–46,900) | 99,100 (87,200–111,500) | ARIMA forecast |
| 2030 | Deaths | 26,200 (21,400–31,500) | 18,600 (14,800–22,900) | 44,800 (37,200–53,100) | ARIMA forecast |
| 2030 | DALYs | 520,400 (438,100–612,300) | 336,900 (278,500–401,200) | 857,300 (724,600–998,100) | ARIMA forecast |
| 2040 | ASIR (/100k) | 3.05 (2.41–3.79) | 2.14 (1.67–2.68) | 2.60 (2.14–3.11) | ARIMA(1,1,1) / (0,1,1) |
| 2040 | ASPR (/100k) | 4.98 (4.12–5.94) | 3.88 (3.02–4.86) | 4.43 (3.58–5.41) | ARIMA(1,1,1) / (0,1,1) |
| 2040 | ASMR (/100k) | 1.84 (1.42–2.31) | 1.52 (1.15–1.96) | 1.68 (1.29–2.11) | ARIMA(0,1,1) / (1,1,0) |
| 2040 | Incident cases | 43,500 (36,200–52,100) | 27,400 (22,800–33,100) | 70,900 (60,000–83,200) | ARIMA forecast |
| 2040 | Prevalent cases | 71,200 (60,100–83,900) | 49,600 (40,300–60,100) | 120,800 (101,400–141,000) | ARIMA forecast |
| 2040 | Deaths | 26,100 (20,800–32,400) | 19,400 (15,100–24,200) | 45,500 (37,900–55,600) | ARIMA forecast |
| 2040 | DALYs | 549,300 (449,600–668,200) | 310,700 (243,800–392,100) | 860,000 (698,400–1,048,300) | ARIMA forecast |
| 2050 | ASIR (/100k) | 3.42 (2.48–4.51) | 2.09 (1.62–2.63) | 2.76 (2.12–3.48) | ARIMA(1,1,1) / (0,1,1) |
| 2050 | ASPR (/100k) | 5.88 (4.72–7.21) | 4.12 (3.18–5.24) | 5.01 (4.02–6.14) | ARIMA(1,1,1) / (0,1,1) |
| 2050 | ASMR (/100k) | 1.82 (1.41–2.28) | 1.43 (1.08–1.84) | 1.63 (1.27–2.06) | ARIMA(0,1,1) / (1,1,0) |
| 2050 | Incident cases | 48,700 (39,200–60,100) | 29,500 (24,100–36,200) | 78,200 (62,100–95,700) | ARIMA forecast |
| 2050 | Prevalent cases | 83,600 (70,100–99,200) | 58,200 (46,800–72,100) | 141,800 (118,900–167,300) | ARIMA forecast |
| 2050 | Deaths | 25,900 (20,100–32,600) | 20,200 (15,300–26,100) | 46,100 (37,400–56,700) | ARIMA forecast |
| 2050 | DALYs | 578,200 (468,300–712,500) | 285,400 (223,600–362,100) | 863,600 (705,900–1,048,200) | ARIMA forecast |

The following table presents numerical projections for gallbladder and biliary tract cancer (GBTC) burden in China for the years 2030, 2040, and 2050. All forecasts were generated using the better-performing ARIMA model (selected based on RMSE/MAE on the 2015–2021 validation set; see Table S1.3).

Prediction intervals (PIs) represent 95% intervals derived from the ARIMA forecasting framework. These intervals quantify the intrinsic uncertainty of a 29-year extrapolation from 32 annual observations.

• ASIR = Age-standardised incidence rate (per 100,000)

• ASPR = Age-standardised prevalence rate (per 100,000)

• ASMR = Age-standardised mortality rate (per 100,000)

• Absolute counts = Incident cases, prevalent cases, deaths, and DALYs

• PI = Prediction Interval (95%)

# **Model Selection Rationale**

For each outcome, ARIMA was fitted independently to the 1990–2021 series. Models were compared on the held-out 2015–2021 validation set using RMSE, MAE, MAPE, and prediction-interval coverage. ARIMA outperformed Prophet on all four metrics for every outcome in China; therefore, ARIMA forecasts are presented in the main text (Fig. 6), and the corresponding numerical estimates are reported above.

# **Caveats on Long-Term Projections**

A 29-year forecast based on 32 annual observations is intrinsically uncertain. The prediction intervals reported above widen substantially for 2040 and 2050, reflecting this growing uncertainty. These projections do not account for potential future disruptions such as large-scale screening programmes, novel therapeutics, or shifts in metabolic risk-factor prevalence. Modelled trajectories therefore represent plausible scenarios rather than deterministic certainties.

Abbreviations: ASIR, age-standardised incidence rate; ASPR, age-standardised prevalence rate; ASMR, age-standardised mortality rate; DALYs, disability-adjusted life-years; PI, prediction interval; ARIMA, AutoRegressive Integrated Moving Average; MAPE, Mean Absolute Percentage Error; RMSE, Root Mean Square Error; MAE, Mean Absolute Error.

Note: PI = Prediction Interval from ARIMA; values in parentheses represent the lower and upper bounds of the 95% PI.
