# Supplementary material for: A cross-sectional analysis and forward projection of China’s gallbladder and biliary-tract cancer burden: Insights from the 2021 Global Burden of Disease Study
Source: Medicine (Baltimore). 2026 Oct 16;105(42):e51008. doi: 10.1097/MD.0000000000051008 (PMC13680927; doi:10.1097/MD.0000000000051008)
Supplement: Supplementary file 3 [file medi-105-e51008-s004.docx]

**Table S3. GATHER Checklist**

| Item No. | GATHER Item | Location (Page / Appendix) | Description / Reported Content |
| --- | --- | --- | --- |
| 1 | Study background and objectives | Page 1 (Title & Abstract); Pages 2–3 (Introduction) | We examined 32-year epidemiological trends (1990–2021) in gallbladder and biliary tract cancer (GBTC) burden in China using GBD 2021 data, and projected incidence and mortality to 2050 via ARIMA and Prophet models. Objectives: characterise age-, sex-, and SDI-stratified burden; forecast future trajectories; inform targeted prevention. |
| 2 | Data sources | Page 3 (Methods §2.1); Table S1 | Country-level GBTC estimates for 1990–2021 were downloaded from the Global Burden of Disease (GBD) Results portal (https://vizhub.healthdata.org/gbd-results/) on 12 March 2024. Data include annual counts and age-standardised rates for incidence, prevalence, mortality, YLLs, YLDs, and DALYs (ICD-10 C23–C24.9). Extraction followed the GATHER checklist. |
| 3 | Data timeframe | Page 3 (Methods §2.1) | Historical data: 1990–2021 (32 years). Forward projection: 2022–2050 (29-year horizon for ARIMA; 29-year forecasts generated with Prophet). |
| 4 | Inclusion / exclusion criteria | Page 3 (Methods §2.1–2.2) | All 204 countries/territories with available GBD 2021 GBTC estimates were included. Three nested cause lists (ICD-10 C23–C24.9 plus histological sub-codes) were reconciled. Counts were rescaled to the 2021 UN World Population Prospects revision. No countries were excluded; data-sparse nations were noted in sensitivity analyses. |
| 5 | Variable definitions | Page 3 (Methods §2.1); Table S1 | Primary outcomes: age-standardised incidence rate (ASIR), age-standardised prevalence rate (ASPR), age-standardised mortality rate (ASMR), and DALYs (sum of YLLs + YLDs), all per 100,000 person-years, standardised to the GBD global standard population. SDI: composite score (0–1) from lag-distributed income, mean schooling years, and total fertility rate. APCs and AAPCs derived via Joinpoint regression. |
| 6 | Data processing steps | Page 3 (Methods §2.2) | Counts were rescaled to the 2021 UN World Population Prospects revision to remove inter-year population drift. GBD-supplied 95% UIs (from 1,000 posterior draws) were retained throughout; all subsequent modelling propagated those draws so final estimates reflect both sampling and modelling variance. No missing-value imputation was required. |
| 7 | Statistical / modelling methods | Pages 3–4 (Methods §2.3–2.5); Table S1 | (1) Joinpoint 4.9.0.1 (NCI): log-linear segments, up to 3 inflection points, permutation test; AAPC with 95% CI. (2) ARIMA(p,d,q): ADF stationarity test; ACF/PACF identification; AICc selection on 70% training split; refit on full series; Ljung–Box residual diagnostics; R 'forecast' package. (3) Prophet: 29-year forecasts, posterior draws (median, 95% UI); R 'prophet' package. Both models implemented independently. Two-sided P < 0.05. |
| 8 | Rationale for modelling choices | Page 4 (Methods §2.5); Page 7 (Results §3.6) | ARIMA captures linear autoregressive structures under stationarity (validated by ADF + Ljung–Box). Prophet accommodates non-linear long-term trends and seasonal components. Both were fitted independently; the better-performing model per sex and indicator (by RMSE/MAE on held-out 30%) was used for the main 2022–2050 projections reported in Fig. 6 / Fig. S1. |
| 9 | Quantification of uncertainty | Pages 4–7 (Results); Figs. 1–7; Table 1 | GBD input: 95% UIs from 1,000 posterior draws propagated through all analyses. Joinpoint: 95% CIs for APC/AAPC. ARIMA: 95% prediction intervals (normal approximation). Prophet: 95% uncertainty intervals (posterior draws). All forecast figures display shaded uncertainty bands. |
| 10 | Sensitivity analyses | Page 9 (Discussion §Limitations); Table S1 | (1) Re-ran Joinpoint with alternative maximum-inflection settings; (2) compared ARIMA vs. Prophet forecasts across multiple train/test splits (60/40, 80/20); (3) tested Prophet changepoint prior scales (0.01–0.5); (4) repeated analyses after excluding data-sparse small-island nations. Core trajectories and sex differentials in China remained stable. |
| 11 | Ethics and data permissions | Page 15 (§7 Ethics Approval) | GBD data are publicly available, de-identified, aggregated estimates requiring no individual consent. The study is exempt from institutional review board (IRB) approval. Data use complies with IHME data-access policies. No clinical trial was conducted (marked 'Not applicable'). |
| 12 | Presentation of results | Pages 4–8 (Results); Figs. 1–7; Table 1; Fig. S1 | Fig. 1: Global/regional age-specific incidence, prevalence, mortality, DALYs. Fig. 2/3: SDI vs. ASIR/DALYs scatter. Fig. 4/5: China age-specific burden by sex. Fig. 6/7 & Fig. S1: 2022–2050 projections for global, SDI-stratified, and Chinese subpopulations. Table 1: absolute counts and ASRs for China 1990–2021. All key findings presented with 95% UIs. |
| 13 | Absolute and relative estimates | Pages 5–8 (Results); Table 1 | Main text reports age-standardised rates per 100,000 person-years (ASIR, ASPR, ASMR, DALYs). Table 1 additionally provides absolute counts (incident cases, prevalent cases, deaths, DALYs, YLLs, YLDs) for China in 1990 and 2021, with PCs and AAPCs for both sexes. |
| 14 | Analysis of heterogeneity | Pages 5–6 (Results §3.1–3.2); Figs. 1, 3 | Heterogeneity examined by: (1) age groups (14 bands, 0–4 to 85+); (2) sex (male/female); (3) SDI quintiles (low, low-middle, middle, high-middle, high) with fixed-quintile assignment; (4) GBD super-regions; (5) China vs. global vs. SDI-stratified aggregates. |
| 15 | Limitations discussion | Page 9 (Discussion §Limitations) | (1) Ecological design precludes individual-level causal inference; (2) findings contingent on GBD 2021 input data quality and modelling assumptions; (3) discrepancies possible with other epidemiologic surveys due to different data-cleaning tools and platforms; (4) disaggregation insufficient to characterise each constituent disease within GBTC individually (GBC vs. CCA subtypes); (5) long-term forecasts subject to unpredictable future interventions. |
| 16 | Comparison with other estimates | Pages 8–9 (Discussion) | Historical estimates are consistent with WHO and national registry data trends. Comparisons drawn with recent GBD-derived publications on GBTC global burden (Lei et al. 2025; Dai et al. 2025). The dual-model (ARIMA + Prophet) cross-validation approach and Joinpoint AAPC provide additional robustness over single-model projections. |
| 17 | Funding and competing interests | Page 14 (§9 Funding); Page 15 (Author contributions) | No funding support for this study (explicitly declared). The funder had no role in study design, data analysis, or manuscript preparation. All authors read and approved the final manuscript. No conflicts of interest declared. |
