# Supplementary material for: A cross-sectional analysis and forward projection of China’s gallbladder and biliary-tract cancer burden: Insights from the 2021 Global Burden of Disease Study
Source: Medicine (Baltimore). 2026 Oct 16;105(42):e51008. doi: 10.1097/MD.0000000000051008 (PMC13680927; doi:10.1097/MD.0000000000051008)
Supplement: Supplementary file 4 [file medi-105-e51008-s005.docx]

Data and R code are available at: https://pan.baidu.com/s/1rMDfXeckYQhX6P8YSqsyGg?pwd=1ad7
